# Establishing a professional learning community for cultivating future design talents using a ‘peer coaching’ mechanism

**DOI:** 10.1016/j.heliyon.2023.e20906

**Published:** 2023-10-13

**Authors:** Li-Yu Chen, Wei-Wei Zhou, Wen-Zhe Hsieh, Rung-Jiun Chou

**Affiliations:** a Dept. of Interior Design, Chung Yuan Christian University, Taiwan; b Dept. of Environmental Design, College of Science and Technology, Ningbo University, China; c Dept. of Landscape Architecture, Chung Yuan Christian University, Taiwan

**Keywords:** Design pedagogy, Design education, Inquiry-based learning, SDGs, Service-learning

## Abstract

The Professional Learning Community (PLC) has an interdisciplinary focus on the humanities, arts, and technology. In order to explore the impact of peer coaching on the learning outcomes of eight university faculty members involved in the PLC for ‘cultivating future design talents through inquiry-based learning’, to develop an innovative approach for the university faculty members professional growth. A qualitative case study approach through six professional development courses with a dynamic revision process, participant observation and in-depth interviews to determine the potential of peer coaching as a tool for PLC teachers. It shows that peer coaching facilitated collaborative learning and positively contributed to PLC teachers' co-construction of knowledge in relation to Inquiry-Based Learning teaching approaches. The study found four processes of course-driven professional development and shows that multiple roles of PLC teachers, the PLC group dynamics, and online peer interaction are important issues in the post-COVID-19 era.

## 1 Introduction

The meaning and function of universities have gradually changed from pure knowledge preservation, pursuit, and creation to close interaction with the world, i.e., the meaning of ‘glocalization’ and the spirit of ‘globalization of thinking and glocalization of action’ [1]. Effective teaching must be forward-looking [2], and teachers must shift their pedagogy to the tomorrow of the child's development [3]. Inquiry-Based Learning (IBL), which fosters positive attitudes towards exploring the unknown, is a necessary pedagogical direction for educating young people for the future [4] and is an important part of building a scientific and cultural community [5] with a deepening understanding, the promotion of learning skills, cross-domain learning, and knowledge construction at the core [6]. Teachers' main tasks are to plan inquiry-oriented activities to help students develop their exploratory and creative skills, form their concepts and cognitions, develop emotions and attitudes, and improve learning outcomes in the process of problem orientation, data search, integration, analysis, evaluation, and problem solving [7,8]. Thus, teachers are an important factor in improving teaching and learning [9], engaging in enquiry-oriented learning entails core research and professional learning skills [10]. Teachers need to continuously develop themselves [11] to improve individual knowledge or skills [12]. Yet professional development activities by traditional teachers do not serve the individual differences in teachers' professional learning and may even be a burden to teachers [13]. COVID-19 was characterized as a pandemic by the Director-General of the World Health Organization on March 11, 2020 [14]. The impact of the pandemic on universities was that campus closures shifted face-to-face lessons to online modes [15]. Systemic disruptions from COVID-19 have transformed the assessment landscape across the world. Alongside repeated shifts to emergency remote teaching [16]. Distance learning became the primary means of delivering instruction for higher education in some art and design classes [17]. COVID-19 has an impact on teacher professional development activities [16], secure and flexible method should be used to comprehensively cope with the complexity of teacher professional learning in varying contexts [16].

‘Peer coaching’ make teachers supporting teachers [18] and can facilitate teachers' self-improvement to meet the needs of learners [19] and can be rooted in pre-service training, where classroom understanding and extracurricular experience are combined to enhance pre-service teachers' confidence in teaching [20]. It is a both horizontal (peer) and vertical (teacher-student) form of cooperative learning [21] which, based on equality, mutual benefit, dialogue, reflection, and practice [22], through reading, demonstration, discussion, observation, and feedback, allows teachers to learn new teaching models from each other, improve existing teaching strategies, and create an environment of two-way communication between teachers and students. ‘Peer coaching’ involves a cycle of objective classroom observations and accurate feedback on teachers' use of new teaching skills [23].

Professional development is essential to the role of the teacher, and has significant implications for lifelong learning for those involved in higher education [24]. The training of teachers can be used to cultivate a culture of lifelong learning, providing a means for improving professional development of the teachers and retaining effective teaching staff over time [25]. There is a clear increase in interest in and recognition of the importance of collaborative and networked learning as professionalisation methods among policy makers and try with forms of collaborative learning such as professional learning communities [26]. Professional learning communities (PLCs) as environments for teacher professional learning Support their professional development activities [27]. PLCs are groups of professionals who learn and grow together, emphasizing different ways of understanding, where teachers, administrative staff, principals, students, parents, and communities maintain a dialogue, collaborate, and share each other's learning experiences [27]. There are papers on the overview of PLCs [28,29] and on the implementation of PLCs and their impact on teachers [30,31], PLCs, as a form of professional development, may help to improve teaching practice, which can ultimately lead to enhancing student learning. PLCs provides an environment for working and learning in collaboration with colleagues. A large body of research points to the importance of teachers working in professional learning communities to implement reformed teaching, as they share knowledge and resources, critically examine and reflect on one another's practices, and use evidence from student work and classroom observations to inform instruction [32]. Many researchers on PLCs focus on the theoretical and conceptual understanding of the PLC ‘construct’ [33] or focused on effects and results of PLCs on student achievements by using quantitative approach [34], the implication on the mental health and learning method of college students [35]. However, few empirical studies on the professional growth and innovative development of PLC teachers using the ‘peer coaching’ mechanism for future talent development during COVID-19. In this research, the case-study university in Taiwan is taken as an example to analyze the detail on the pressures faced by teachers in their growth process, Research into the factors that may influence such pressures and how teachers working in professional learning communities changed during COVID-19, In short to explore an innovative approach to PLCs that aims to promote the professional growth plan of PLC teachers for the cultivation of professional talents, and to complement the existing literature on PLCs.

## 2 Overview of PLCs at the case-study university

Since 1989, the case-study university has been pursuing the concept of ‘holistic education’, emphasizing the balance between specialization and general education, between learning and personality, between the individual and the community, and between body, mind, and spirit. In recent years, the school's educational philosophy has been highlighted by exploring IBL, which has been implemented with good results so that the teachers are enthusiastic about professional growth. The university's PLC adopts a ‘peer coaching’ mechanism. It takes IBL as a pathway, combines forward-looking issues and altruistic practices, makes the SDGs of the United Nations a direction, acts on the learning goal of addressing local or community issues, and maintains a high degree of consistency with local future development and related policies.

**Fig. 1:**
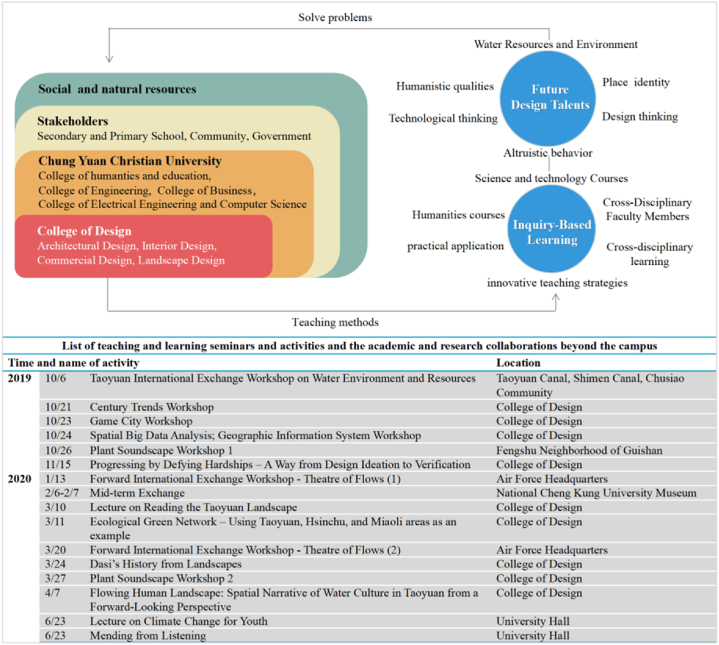
The relevance of cross-cultural, cross-knowledge and cross-border curriculum planning and teaching activities.

The professional development of teachers, the innovative development of higher education, and the progress of society and economy cannot be separated from the tripartite cooperation among universities, industries, and the government, so as to realize the synergistic development through win-win cooperation, resource sharing, and complementing each other's advantages [36,37]. The case-study university focusing on water resources issues, it invites experts, scholars, and educators with practical experience to hold seminars and invites stakeholders, including the local government, a local senior high school, and many other entities to conduct off-campus academic and research collaborations in search of forward-looking issues and industry-school cooperation related to the local water environment. Based on this, a course cluster on local environmental issues can be formed and extended for the exploration of international environmental issues and models of problem solving. The aim is to develop the professional growth of PLC teachers for cultivating future talents and facilitating the integration of teachers by analyzing the correlation between professional subjects (Fig. 1).

Cross-discipline course electives collaboration with a local senior high school, forward-looking design teachers from four departments in College of Design, taking high school students through different aspects of design. The course ability direction is to learn about the relationship of design in the human race, things, objects, life, and the surroundings from a design-relevant perspective, as well as sustainable evolution. Through the study to understand the importance of human-centered design, learn to think through different perspectives and multiple ways of thinking, combine theories and methods, and think about how to make decisions. Exciting to see that new schools wish to join PLC so that a wider inter-campus network is expected in the future.

This study echoes [38] that consulting with local schools is ways teacher educators can demonstrate relevance and responsiveness to the local culture. Blending academy and field-based cultures could provide deep learning for teachers.

## 3 Methods

### 3.1 Research design and methods

FOR Research design: The study adopts a single-case design in which the interactions of people and events that occurred within the topic are considered as a unit of analysis, and the logic among the data is emphasized through cross-analysis of multiple and rich research data [39]. In this study, eight faculty members with more than 10 years of teaching experience, doctoral degrees from domestic and international universities, and a familiarity with the study process were selected. A one-year (2019–2020) case study was then conducted to observe the PLC for the eight teachers on ‘inquiry-based learning for future talent cultivation’ utilizing the ‘peer coaching’ mechanism, and to analyze their reflections and the changes involved in the process of growth. The demographics of interviewees as in Table 1.

**Table 1:** Demographics of interviewees.

ID	Academic Qualifications	Teaching Experience		Participation
Teacher A	Art and Creative Practices, PhD	more than 10 years	Scene and Atmosphere Creation, Curatorial Studies, Material Aesthetics Research and Development, Art Practice	‘Architecture and Plants’ course taught by teachers from the Department of Architecture and French sound artists
Teacher B	Planning and Landscape, PhD	more than 15 years	Landscape Planning and Design, Urban Water Environment Management, Social Landscape Architecture and Development	Geographic Information Systems (GIS) and Future Vision Applications
Teacher C	Architecture, PhD	more than 30 years	Taoyuan Water Resources and Environmental Control Study, Community Building, Preservation of Settlements and Redevelopment of Historic Districts, Architectural and Environmental Design, Social Design	Climate Change and Sustainable Water Environments
Teacher D	Comparative Literature, PhD	more than 10 years	Comparative Literature, Contemporary French Literature, French Art Studies, Discrete Discourses, Intercultural Studies	Contemporary Literature and Ecology
Teacher E	Visual Communication, MA	more than 10 years	Cultural and Creative Design	The Study of Cross Media Design
Teacher F	Business, PhD	more than 10 years	Branding, Advertising Strategy, Integrated Marketing Communications, Marketing Aesthetics, Design and Marketing	Design Industry Ecosystem
Teacher G	Engineering, PhD	more than 10 years	Ecological and Environmental Engineering, Fluid Mechanics in Civil, Hydraulic and Environmental Engineering	Ecological Engineering
Teacher H	Engineering, PhD	more than 10 years	Interior Design, Architectural and Urban Design, Digital Space Design and Theory	Urban Space Simulation

Before conducting the participant observation, the researchers explained to the teacher the purpose and mode of participation in the observation. An observer and an interviewer were used to observe and interview the same phenomenon or behavior in order to understand the degree of agreement between the observation and the interview.

The in-depth interviews were conducted by the researchers after the teachers had voluntarily signed an informed consent form. This research process adheres to the principles of voluntariness, confidentiality, and anonymity.

The second semester influenced by Covid-19, video and audio communication features need to be considered, the main interview tools are TEAMS, LINE, email. The interviewees were given the choice of whether or not to turn on their camera in interviews, a separate computer device was used for recording with the consent of the interviewees. The interviews lasted between 25 and 40 min, and all interview videos, audio, transcripts, emails, and social software chat records were not shared with any third party and were deleted immediately after the study was completed.

FOR ethical consideration: As the interviewers were colleagues who knew each other and belonged to a homogeneous group (Shamdasani & Stewart, 2015), the researchers not only emphasized the importance of confidentiality to the members before the interview, but also used interview techniques to encourage each participant to speak up and express different opinions in order to minimize the influence of interpersonal interactions on the content of the interview discussions, such as opposing views or minority ideas that could not be easily voiced in the group [40]. This research process adheres to the principles of voluntariness, confidentiality, and anonymity. The interviewees read and agreed to the consent form and the information sheet before the interviews. Transcribed manuscripts were given to the interviewees to modify and supplement.

FOR PLC overview and implementation: The year-long professional development program was designed with seven 3-h group courses, presenting a comprehensive approach to problem solving and professional dialogues and reflections on teaching and learning. In addition to the informal interviews in the professional development courses, three individual in-depth interviews (each of at least 35 min), five follow-up interviews, and three group interviews were conducted with each participating teacher.

FOR Data collection and analysis: Qualitative study is a process of reflectivity [41] and is about using a variety of data collection methods to improve the ‘quality and rigor’ of a study [42]. Therefore, triangulation was used to obtain rich data and to collect observations, interviews, course materials, and other data. The process of thick description and interconnecting interpretation is used to present the feelings and experiences of the subjects in a complete and realistic way through graphics and text. In addition, participant observation was used to study the learning process of teachers in the PLC implementation process, to complete the research teams notes and reports, providing reflections and insights. Noteworthy, a number of respondents continued to provide additional information through the LINE software program after the interviews had been completed, as time passed and the format of teaching and learning changed as a result of COVID 19. These data sources added to the richness and accuracy of the study.

### 3.2 The six professional development courses with a dynamic revision process

Initially, the research team went into the classrooms to observe courses and reflect on the organization of the professional growth program. A research team members reflection note:

Through the course observation, we found that the teachers were already familiar with the inquiry-based curriculum, but how to develop a cross-disciplinary issue framework and how to integrate future talent cultivation into the inquiry-based learning are the key. (2019Oct12).

Therefore, the first professional development course, entitled ‘listening and observing’, is the starting point for teachers' professional growth to complement teachers' professional knowledge, to construct an interdisciplinary framework of issues, incorporating future talent development into IBL.

By listening to teachers' needs and observing their teaching habits for understanding the educational concepts, methods and skills in the teaching process, it provides them with professional knowledge to supplement their deficiencies, including up-to-date subject matter knowledge, educational psychology and teaching skills, establish a framework for interdisciplinary issue.

**Fig. 2:**
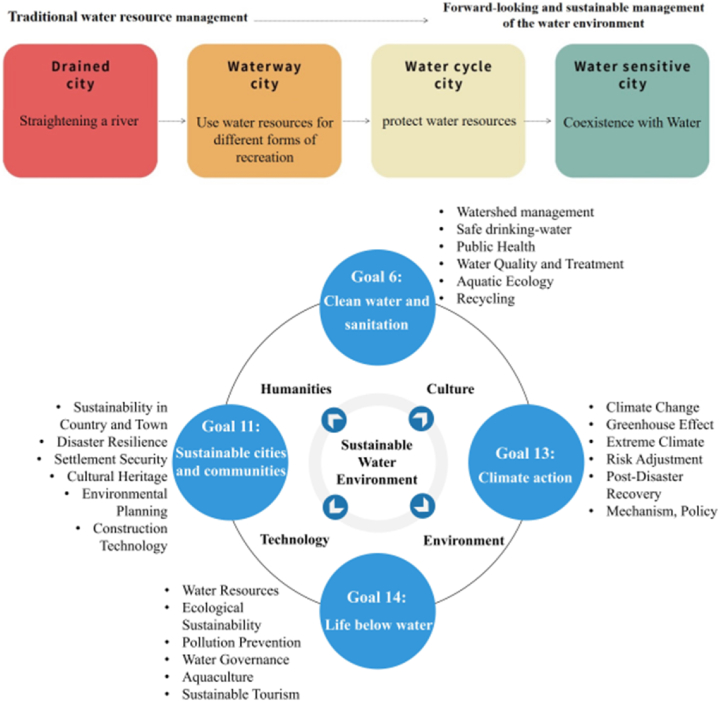
Cross-disciplinary issue framework in response to SDGs.

Water space carries the development of human civilization. However, as water resources are threatened by depletion and environmental factors such as climate change, traditional models are no longer sufficient to deal with the complex problems associated with the water environment in the future, and it is necessary to consider the multiple roles of water in urban public space and move towards water-adapted cities. Therefore, Teachers, education experts, and stakeholders could collaborate in establishing cross-disciplinary issue frameworks by using a ‘peer coaching’ mechanism that links four of the Sustainable Development Goals (SDGs), e.g., Goal 6 Clean Water and Sanitation; Goal 11 Sustainable Cities and Communities; Goal 13 Climate Action; Goal 14 Life below Water, as show in Fig. 2. The framework helps teachers to incorporate future talent development into IBL, integrate knowledge and skills from different disciplines, then propose teaching programs that closely follow the SDGs, present relevant problems and corresponding solutions in the teaching and learning process, thus cultivating teachers' ability to solve complex interdisciplinary practical problems.

By integrating science, art and design, since experts in different fields could work together to discuss and solve problems by creative and innovative solutions, thus achieving mutual support and promotion of theory and achieve the goals of Collaboration, Humanization, Empathy, Ecology and Renaissance [43]. The convergence of Denology (Design Science and Technology) and Humart (Humanities and Arts) implies collaboration and exchange among the fields of science, technology, art and design for generating innovative thinking and interdisciplinary collaboration in the benefit of economic development [44]. Therefore, the combination of humanities and technology may show a unique charm in the field of art and design. The first professional development course focuses on combining 'humanities' and ‘science and technology’ into art practice and considers the use of everyday materials to develop future design concepts, e.g., energy collection from garbage cans, plastic bottle gardens, cardboard furniture and wearable waste products. It combines waste disposal with renewable energy, advocates environmentally friendly lifestyles, fosters problem-solving skills, interdisciplinary thinking and innovative thinking among the students, so as to comply with the future development needs. Two teachers said that:

It is challenging to imagine the future urban environment from everyday materials. (2019Nov02 - Teacher A).

We have overlooked the importance of seeing what people don't do and listening to what people don't say, leading to a lack of exploration and discovery, which can be a barrier to innovation. (2019Nov02 - Teacher D).

In short, by listening to and observing teachers' teaching habits, supplementing professional knowledge, framing interdisciplinary questions, and integrating future talent development into inquiry-based learning, the first professional development course provides teachers with targeted and innovative instructional design and practice.

The second professional development course, entitled ‘awakening to action,’ focuses on helping teachers explore the importance of social welfare, understand the ethical considerations of design ethics, and awaken empathy in an interdisciplinary context. Discussions on social well-being will help teachers recognize their roles and responsibilities, encourage them to reflect about how to provide support and assistance to disadvantaged groups by using their professional knowledge and skills, then cultivate a sense of social responsibility and civic-mindedness among students [45]. Relevant discussion of design ethics helps teachers to realize the potential impact of teaching activities on students and teach students how to introduce ethical principles and values in the design process [46]. Empathy is an ability to understand and feel the feelings of others, which guides teachers to explore the concepts and methods of empathy, encourage them to apply empathy in teaching practice, so as to build the effective relationship between teachers and students [47]. With the second professional development course, it awakens teachers' cognition of the social significance and ethical responsibility of education work, then establishes a closer relationship with students.

The third professional development course focused on summarizing the connotations of design thinking for future talent and innovative strategies for IBL proposed by experts and scholars, with an emphasis on the integration of pragmatism and technology, as well as the pulse of student observation and inquiry. Taking the course, ‘Geographic Information Systems (GIS) and Future Vision Applications’ as an example, the teacher introduced the new technology of spatial big data software, allowing students to explore how the geographical environment changes from the vertical timeLINE of historical comparison to the horizontal human and social and economic development of the community, exploring the changing process of environmental energy, and increasing new knowledge on understanding and constructing. This course combines technology to practice. In this study, the third development course is named as ‘pragmatic and practical’.

The fourth professional development course was the ‘retrospection and reflection’. This involved collecting teachers' lesson plans, analyzing the elements of the lesson plans, the flow of activities, the content of the concept of future talent cultivation, the orientation of inquiry, and understanding teachers ideas about the content of the course and what they had gained. With a professional dialogue about practice, sharing, and review, and a cycle of reflection and action, teachers are more capable of changing and developing their course and then teaching it with confidence.

The most rewarding thing is how I can use cross-disciplinary collaboration, combine technology and art, and internalize these abilities to explore more materials or opportunities. (2019Dec27 - Teacher A).

We need to be sharper in gathering more material from everyday life that can deepen students' understanding of the field, stimulate imagination, and learn to use technology to interpret ideas. (2019Dec27 - Teacher B).

From an altruistic point of view, combined with practical work, we focus on students understanding and application of professional ethics, recognition of social responsibility, and respect for diverse perspectives. (2019Dec27 - Teacher C).

For online courses, the five PLC teachers (Teacher D, E, F, G, H) all mentioned the defects of online teaching. For example, online learning is often incoherent and poor thinking due to equipment problems, signal problems, listening rhythm problems (such as not understanding, not keeping up). It is difficult for the coach to receive feedback information in the first time. They still proceed at the same pace, resulting in some PLC teachers failing to meet meet the standard of learning.

It was difficult for teachers to achieve sufficient and enthusiastic interaction in the limited time available through online communication (2019Dec27 - Teacher D).

Interactive activities in online classes become more difficult due to the time delay, with a significant decrease compared to face-to-face instruction. (2019Dec27 - Teacher E).

Coach spends more time talking to PLC teachers because it is more difficult to communicate on TEAMS (2019Dec27 - Teacher F).

Before the COVID-19 epidemic, case sharing and seminar activities are available in the seminar room, we could ask about each other's learning progress and share our experience and harvest, which is a good way for me to learn. But such activities have decreased because of the pandemic (2019Dec27 - Teacher G).

I feel isolated in the asynchronous courses，online communication is difficult when there are differences of opinion with peers in different fields (2019Dec27 - Teacher H).

Furthermore, teachers reported that developing inquiry skills for students at different levels is worthy of further discussion. Studies have shown that watching videos of others teaching helps to facilitate deeper reflection, and triggers higher levels of emotional and participatory motivation in both the viewer and the viewed [48]. As a result, the research team developed the fifth professional development course, entitled ‘lesson observation and review’ to help teachers organize their teaching activities after observing them in class, and filmed videos of different classes conducting teaching-related activities. With the fifth professional development course, peer lesson observation and reflection were conducted, and the course invited scholars of design education to share innovative approaches to the cultivation of future talents and the design of teaching and learning for the development of students' inquiry skills at different stages. The professional development activities based on ‘lesson observation and review’ are important pillars for teachers to actively build professional knowledge for inquiry-based curriculum development and teaching practice.

In the second half of the semester, due to the impact of the school closure of the epidemic, the use of the Internet platform and Facebook social media helped to extend the physical space, to make up for the lack of face-to-face professional conversation, allow members to continuously communicate and exchange information and contact, and reduce the communication barriers and barriers caused by the school closure of the epidemic. However, teachers found that during the COVID-19 pandemic, college students were facing a lot of future-oriented pressures, e.g., worries about degree delays and concerns about future employment prospects. Therefore, from the sixth professional development course, ‘awareness and refinement,’ teachers could engage in specific curriculum revision and redesign, followed by further curriculum revision and trial teaching, then incorporate socially responsible and community-centered teaching and learning activities through the Internet networking platforms and community organizations, thus introducing community resources and hot social issues to enrich class teaching. This active social engagement may not only moderate the relationship between students' academic stress and well-being, but also endow teachers a profound understanding of the significance of ‘Cultivating Future Design Talents through Inquiry-based Learning’.

**Fig. 3:**
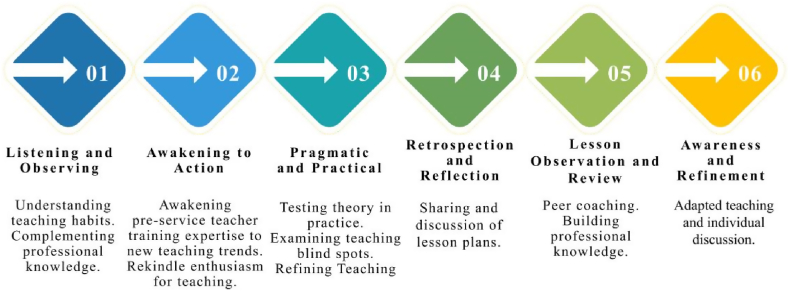
The six professional development courses with a dynamic revision process.

Due to the complexity and variability of the ‘school’ context, a theoretical framework is needed to examine the long-term development of PLCs [49]. As shown in Fig. 3 about the six professional development courses with a dynamic revision process, it is found that teachers were mostly lively and creative, but confused about how to choose curriculum topics and how to introduce forward-looking design thinking into IBL. After interviewing the teachers, it is found that the professional development courses are not a top-down model, nor is it a one-way or static activity, but should be planned according to the needs of teachers and the development of students' inquiry skills, and should consider the links between theory and practice. Therefore, case sharing and seminar activities are more preferable to help teachers identify the strategies and practices of IBL for the cultivation of future talents and to form a dynamic revision process of professional development, where teachers as the main focus.

## 4 Results

### 4.1 The four processes of course-driven professional development

**Fig. 4:**
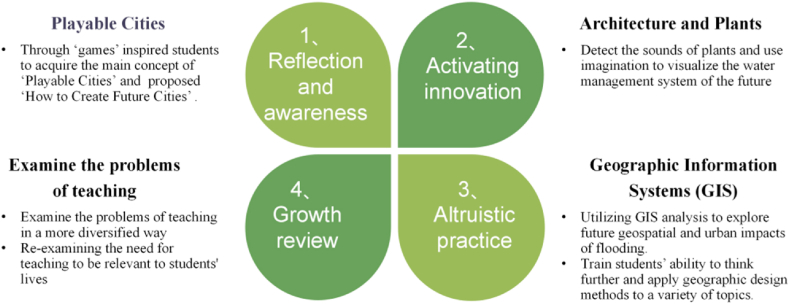
The four processes of course-driven professional development.

The four processes of course-driven professional development as show in Fig. 4.

The first process of course-driven professional development is reflection and awareness. Design thinking is increasingly seen more as a way to build the capacity of 21st century students and provide them with a foundation to effectively address the changing challenges facing the global society of tomorrow [50]. By introducing topics related to future fields, disciplinary barriers can be reduced and interdisciplinary collaboration facilitated [51]. The first professional development course introduces how IBL combines humanities and arts to guide students to develop their imagination of future cities from everyday materials (games). The teachers found that ‘games’ are often used as a teaching medium to enhance the class vibe, but rarely have a connection to the future talent development. So, in addition to lectures and discussions, a cross-media designer was invited to conduct a workshop educational activity on the theme of ‘Playable Cities’ by transforming ‘urban exploration’ into a ‘gaming experience’. Taking the cross-discipline, masters course ‘The Study of Cross Media Design’ as an example, a local town is selected, and the humanities and natural environment and environmental changes in the area are used as the text to complete a ‘Future City’ App with multiple media content. The entire teaching process involves inquiry-based teaching. First, students are inspired to acquire the main concept of ‘Playable Cities’ through ‘games’ and then the ‘How to Create Future Cities’ questions are proposed. Then the students are asked to choose a town to conduct a landscape survey by starting from what they see, hear, and the surroundings they touch, and then entering the flowing state of the local landscape. They accept the parameters of substance flow, information flow, and material flow in the environment of concern through the senses, then create different scenarios in the virtual space, by combining VR and AR technologies, participants may play different roles such as city planners, businessmen and citizens while interacting with other players, observing the behaviors of different groups, discovering the expansion and interaction of different fields, and obtaining new design ideas and thinking directions.

In the past, teachers mostly started directly to ideate and to develop a program but neglected the ‘orientation’ stage. Only by allowing students to go into the field and gather clues from the environment and the voices of users can they accurately carry out ‘orientation’ and build Meta-cognition [7].

A group interview about the future talent development concepts shows that:

Moderator: Let's discuss what the hidden meanings of future talent development are in the ‘Playable Cities’?

Teacher C: Observe the people and things in the city, combine VR, AR, and make the city playable … Exploring the city from the perspective of games, gaining new ideas and thinking directions, discovering the extension and interactivity of each area, triggering new ideas and creating more innovative models for the benefit of society, right?

Teacher E: ‘Playable Cities’ is a new philosophy that makes people think about how to create cities of the future … Make the combination of technology and the arts as a means to stimulate more forward-thinking ideas for teaching and learning.

The second process of course-driven professional development is activating innovation. Using the course of ‘Architecture and Plants’ as an example, it requires forward thinking and perspectives through plant sound detection, and requires a network of cross-disciplinary inquiry learning which is based on care for nature and the environment and design ethics. Technology is used in practical work to reveal hidden biological messages and to map and create new areas for the future.

This course is co-taught by teachers from the Department of Architecture and French sound artists. The course uses ‘sound’ as a way to ask a series of questions about the ‘messages of the plant world’. Students listen to the fluids and energy in the ecosystem, imagine the operation of the whole system; think about the connection between architecture, nature, and the environment; generate their own ideas, and then form a discussion and create a 1:1 model of a Green House. IBL is a successful strategy for improving student's skills in analyzing, judging, and using aesthetic and contextual knowledge in writing [52], so the final task of the course is assigned by asking students to integrate the process and results of their inquiry in writing, with the text published in print or online, or presented as a read-aloud audio file.

About the process of activating innovation, two teachers said that:

Through cross-pollination between text, video, design and other disciplines that cross boundaries, new ways of seeing can be facilitated. (2019Nov12 - Teacher C).

A holistic understanding of the landscape comes not only from the visual, but also from a combination of other senses, life experience, and knowledge. Through the media of photography, text, catalogues, cross-sections, and aerial views, we present the interaction between everyday landscapes and natural events, not only presenting the past of the site but also projecting its future. (2019Nov12 - Teacher E).

A group interview about the process of activating innovation shows that:

Teacher G: Students see what they see through the process of free inquiry, and it is more important for them to explore firsthand than to be instructed by teachers.

Moderator: How is this different from traditional teaching?

Teacher F: Discovering rules and developing competencies through inquiry is essential to building an inquiry-based learning network.

Teacher H: Designers should not only be able to solve problems, but also be able to ask questions, especially in response to future industrial and environmental changes.

For the video course, the three PLC teachers（Teacher B, D, E）mentioned that one of the benefits of this video class is that there are chat records to review and consolidate after class. Therefore, the effect of learning is better than before, because they can use the fragmented time to review anytime and anywhere, improving the learning efficiency, and having a more comprehensive understanding of the knowledge. Instead, the respondent mentioned that the increased work challenges due to COVID 19 plague teachers and caused them anxiety and irritability.

Teacher B: It is difficult for me to spend more time preparing lessons before, because I need to spend on administrative time, teaching time, learning and service. However, In our conversion to online courses during the COVID-19 epidemic，When I don't understand something, the coach can text me in the chat bar of TEAMS, I can Watch the replay and save chat records, review them anytime and anywhere after the lesson.

Teacher C: Teachers only need to be responsible for their teaching task before the COVID-19 epidemic, but the teacher needs to be responsible for more and more things now, such as prepare for the new semester teaching, responsible for collecting the students' travel trajectory information. It is difficult for me.

Teacher D: In the seamless switch between the two identities of teachers and volunteers, the teacher becomes an ‘all-around soldier’, seize a ‘fit’ time into learning, not only let me realize the true meaning of education, but also make me more cherish every minute of the class, follow the pace of the teacher's pace, concentrate, actively interact, and take notes carefully.

Teacher E: With the reduction of face-to-face class and the delay of audio hindering the effective interaction, I have focused on cultivating my sight-reading ability.

The third process of course-driven professional development is altruistic practice. Taking ‘Geographic Information Systems (GIS) and Future Vision Applications’ as an example, it aims to train students ability to think further and apply geographic design methods to a variety of topics. Given a practical project, students work on GIS to complete analysis, planning, and design. Three teachers said that:

Local identity is created through IBL, design thinking is used to think about solutions to problems, and through GIS, students develop the deep and broad professional knowledge and skills that will serve as the basis for their future. The entire structure is designed to serve society and promote altruistic practices. (2019Nov20 - Teacher A).

Cross-disciplinary learning allows us to break through existing thinking, to add new ideas to existing ones, and to apply what we have learned to form a new set of own opinions. (2019Nov20 - Teacher E).

Through altruistic practice, the students can remember the relationship between social design and public participation … Plant the seeds of becoming a quality designer in the future in their minds. (2020Jun27 - Teacher H).

The fourth process of course-driven professional development is growth review. The complex problems facing the world of the future are beyond the reach of a single designer and require a cross-disciplinary, cross-cultural, cross-knowledge, and cross-boundary perspective and scope [53]. IBL can find possible solutions to complex and fluid foresight issues through interconnectedness and collaboration. Teachers are re-examining the need for teaching to be relevant to students' lives. About the process of growth review, three teachers said that:

It's easier and more engaging when it's relevant to students' lives. (2020Jun27 - Teacher A).

Students should be given more time to try and build concepts even if they are wrong. (2020Jun27- Teacher E).

Often I should take a step back and let the student figure it out. (2020Jun27 - Teacher G).

From the above, it is found that teachers are willing to examine the problems of teaching in a more diversified way, and that IBL should follow the development of student's interest and ability to inquire and should consider the connection between theory and practice.

## 5 Discussion

Fostering the development of Professional Learning communities (PLCs) should be a priority for education because of their capacity to enhance teacher's professional development [54]. Effective professional development is combining working and learning, collaborative and collegial, practice oriented are key features of a PLC [55]. Literature review indicates few empirical studies on the professional growth and innovative development of PLC teachers using the ‘peer coaching’ mechanism for future talent development during COVID-19. This research establishing a Professional Learning Community for ‘Cultivating Future Design Talents through Inquiry-based Learning’ using a ‘Peer Coaching’ Mechanism, to develop an innovative approach for the university faculty members professional growth.

### **5.1** LINE, TEAMS courses and asynchronous video courses combining working and learning

Participant observation shows that teachers in PLCs play many different roles, i.e. serve different departments and institutions located in different places, making it more difficult to connect, and requiring extra time and effort to participate. In addition, the teachers usually spend so much time on their administrative duties, teaching hours, study, and service that they may not be able to allocate time for the initial planning of a course that requires members of multiple faculties to prepare and discuss. It may be difficult to get teachers to spend more time planning courses, or to communicate with each other when there are differences of opinion between them in different areas. Covid 19 changes the working and learning environment during school closureshas, led to a change in peer Co-teaching strategies, PLC coaches change online courses by sending materials via distance learning platform, teachers connect with each other through TEAMS or LINE, feeling of isolation had a great impact in the asynchronous course. The previous comments show that TEAMS courses and asynchronous video courses have their limitations, however, on the other hand, Use TEAMS and asynchronous video with PLC teaching enhanced awareness of corresponding stress and self-adjustment, additional stress and adjustment of psychological state, motivate PLC teachers to change their approach to autonomous learning, such as learning from video recordings or course replays, review them anytime and anywhere after the lesson. Cultivating sight-reading ability, promoting the ability of comparing and analyzing, as well as self-examination skills, doing more research, reading more. The study echoes [56] that teachers experiences and beliefs had little impact on their distance learning practices, while confidence in using digital technologies increased significantly during the lock down in the COVID-19 epidemic. However, different levels of participation may have different impact on PLC teachers and produce differences in learning outcomes and personal development.

However, new evidence suggests that the effectiveness of various distance learning interventions has been limited. Nonetheless, this study builds on decades of evidence on ‘inquiry-based teaching’ and peer-to-peer teaching, innovatively combining past evidence with current contexts to identify what works, while innovatively tailoring telephone coaching to the learning environment of PLC teachers (PLCs in epidemics). In this study, coaches sent weekly text messages and provided voice coaching to PLC teachers via a LINE group, which led to improvements in teachers' learning (the learning environment for PLC teachers changed dramatically during the epidemic, as they no longer sat in classrooms and listened to lessons, but received text and LINE telephone voice coaching at home). Tutorials for a total of eight weeks. This intervention is not only effective, but also very cheap and cost-effective. Simply offering LINE to help with teaching is not entirely effective; it is important to provide lively lesson coaching directly to PLC teachers via mobile phones. One reason why the successful implementation of distance learning via LINE has been so effective is the high coverage and low cost of mobile phones.

### **5.2** External or internal coaches’ collaborative

There are both active and inactive members of the community [57]. The actual activity of co-preparation and co-teaching is influenced by the extent to which each member participates, and different levels of participation may have a negative impact on other teachers and produce differences in learning outcomes and personal development (Prenger et al., 2019). This is echoed by the study that some teachers are simply passive in waiting for others to respond during teamwork activities. However, other teachers felt that they could learn more from taking on the extra workload. This study echoes [58] that peer coaching is associated with team learning beliefs and behaviors. It is therefore the responsibility of the leader to manage teachers' motivation and engagement, for example, by giving passive teachers important tasks and giving them more guidance on what to do and how to do it, in order to maintain the group dynamics. This, along with viewing mentors as ‘cognitive coaches’ [59], ensures that teachers are able to collaborate and solve problems. But these coaches are either external experts or peer teachers that guide the team from within. The current literature is in debate on whether external or internal coaches are the most effective in supporting for teacher professional development [58].

There are both external and internal coaches in this study. External coaches enrich the team learning process by bringing in curricular design expertise, pedagogical content knowledge, or knowledge on the reformed curriculum [60], internal coach tends to be a peer teacher who is a full member of the team, guiding it from within [61].

In this study, PLC prioritized individual traits and engagement of participating teachers. The pace of the epidemic online learning between teachers and students is different, as it is often incoherent and poor thinking due to equipment problems, signal problems, listening rhythm problems. It is difficult for the coach to receive feedback information in the first time. They still proceed at the same pace, resulting in some PLC teachers failing to meet the standard of learning. In this context, the coaches implement different levels of training programs for teachers at different levels of engagement and provide directional guidance to improve the teaching effect rather than using a didactic curriculum suitable for all people. In addition, the implementation of different levels of training programs for teachers at different levels of engagement is also necessary for effective learning and to avoid undermining teachers' confidence and interest in learning.

### 5.3 Constructing a co-learning imagination that mediates between virtual and real boundaries

In the first semester，coach choose the power of ‘flick push’ to strengthen the action choice of members by the collective activities of teaching inquiry. Community operation contains fixed of teaching inquiry activities, mainly prepared, class and class discussion, preliminary planning and final review. The level of interaction and the richness of the activities of the teachers in PLCs are adversely affected by the COVID-19 pandemic. Participant observation revealed that it was difficult for teachers to achieve sufficient and enthusiastic interaction in the limited time available through online communication. Therefore, it was switched to an intensive synchronous online peer coaching approach that makes use of holidays to improve learning efficiency, as in Table 2.

**Table 2:** Intensive synchronous online peer coaching approach that makes use of holidays.

International Online Special Lectures				
University	UCSI University of Malaysia		Chung Yuan Christian University, Taiwan	
Participants	2 teachers and 20 students		2 teachers and 20 students	
Groups	Team A: 1 teacher in Taiwan, 5 Malaysian students and 5 Taiwanese students	Team B: 1 Malaysian teacher, 5 Malaysian students and 5 Taiwanese students	Team C: 1 teacher in Taiwan, 5 Malaysian students, 5 Taiwanese students	Team D: 1 teacher in Taiwan, 5 Malaysian students, 5 Taiwanese students
Coteaching in International Contexts				
	International Design Workshops		Holiday Experience Camp	
Teachers	2 teachers in Chung Yuan Christian University, Taiwan		2 teachers in Chung Yuan Christian University, Taiwan	
Course	International Thematic Design Practice		Creative Living Landscapes	
Participants	20 students in UCSI University, Malaysian and 20 students in Chung Yuan Christian University, Taiwan		21 overseas students across Chung Yuan Christian University, Taiwan	
Organizers	USR Task Group		International Division + USR Task Group	

This approach includes International Online Special Lectures, the organization of International Design Workshops and Holiday Experience Camp in conjunction with multinational teachers, stimulating deeper discussions using different textual, linguistic and visual media expressions and complementary responses such as drawing, writing and communication. In order to increase peer exchange opportunities using a variety of interactive learning formats. All topics related to teaching and practices were included in the interactive discussions.

Intensive synchronous online peer coaching approach promotes teacher's collaboration and professional development, which echoes [62] that collaborative processes enabled teachers to access tacit knowledge resources, this both challenged and informed teacher beliefs, motivating joint development of enhanced practices.

In addition, in order to make up for the lack of face-to-face professional dialogue, In the second half of the semester，the use of the Internet platform and Facebook social media helped to extend the physical space, allow members to continuously communicate and exchange information and contact, Share experience online or participate in online seminar, and reduce the communication barriers and barriers caused by the school closure of the epidemic, Constructing a co-learning imagination that mediates between virtual and real boundaries.

This study echoes [63] that learning interactions need to be diverse and open to all aspects of daily activity, with a variety of interactive content, expressions, and responses. The study shows that intensive synchronous online peer coaching was effective in increasing the extent of peer interaction. This study supports the suggestion [64] that more effort is needed to effectively deliver intensive online learning, including synchronous online interactions between students and teachers, compared with extensive online learning focusing on compiling digital self-study resources.

Peer coaching activities correlate with the team effectiveness and the quality of material developed [58], In order to improve the quality of material developed, ‘games’ are often used as a teaching medium in PLC educational activity in our study, help to enhance the atmosphere of the classroom and provide images of activities which are well rooted in contemporary cultures and which plausibly enter into learning processes that go beyond specific narrow skills. Games stimulate the imagination and gives these activities their quality of learning-richness. This study echoes [62] proposal that，In order to comprehensively cope with complexity of teacher professional learning in varying contexts, imagination could be used to develop practice knowledge.

Many teachers and educators when talking about the nature of their profession describe it as a mixture of art and science [65]. Art allowed time to think, to dream, to gaze, to get a new idea and try it and drop it or persist, time to talk, to see other people's work and their reaction to yours [66]. Our research establishing a Professional Learning Community for ‘Cultivating Future Design Talents through Inquiry-based Learning’ using a ‘Peer Coaching’ Mechanism, focuses on art and science, the practice of art through the combination of 'humanities' and ‘technology’, considered how to develop future design concepts.

## 6 Conclusions

This one-year (2019–2020) case study is designed to analyze the experience of eight university faculty teachers who participated in a PLC for ‘cultivating future design talents through IBL’ using a ‘peer coaching’ mechanism. The study indicates that the six professional development courses with a dynamic revision process include listening and observing, awakening to action, pragmatic and practical, retrospection and reflection, lesson observation and review, and awareness and refinement. The four processes of course-driven professional development include reflection and awareness, activating innovation, altruistic practice, and growth review. It shows that multiple roles of PLC teachers, the PLC group dynamics, and online peer interaction are important issues in the post-COVID-19 era. PLCs emphasize the uniqueness and specialization of different fields, and functions as an integrated, cross-disciplinary, diverse, autonomous, and resilient institution to deepen education and expand impact, and to achieve the long-term benefits of cross-disciplinary learning. The sustainability of PLCs is dependent on campus culture [49], strong inter-campus partnerships, shared goals and resources [9], communication and continuous reflection [28]. The current study has some limitations, including the number of participants was low and the duration of the study was short, and the fact that it was conducted at a single-case design. On the other hand, long-term follow-up for PLC teachers' practice is needed for future research. In the future, it is needed to connect more with professional communities abroad, increase participation in more challenging and interactive forms of professional learning, learn international tools for the innovative design and R&D of foresight issues, build a knowledge base of foresight issues, thus internalizing and forming our own methodologies, producing different forms of knowledge derivatives, combining the results with presentations to implement localization of PLCs and integrating technology into the future talent training courses.

## Ethical considerations

All procedures performed in the study involving human participants were in accordance with ethical standards. Oral informed consent was obtained from all individual participants included in the study.

## Funding information

The study was funded by University Foresight Education Project (B-054-108-1-0542), 10.13039/100010002Ministry of Education, Taiwan.

## Data availability statement

Data will be made available on request.

## CRediT authorship contribution statement

**Li-Yu Chen:** Conceptualization, Formal analysis, Funding acquisition, Investigation, Methodology, Writing – review & editing. **Wei-Wei Zhou:** Conceptualization, Formal analysis, Investigation, Methodology, Writing – original draft. **Wen-Zhe Hsieh:** Conceptualization, Formal analysis, Investigation, Methodology, Writing – original draft. **Rung-Jiun Chou:** Conceptualization, Formal analysis, Funding acquisition, Investigation, Methodology, Writing – review & editing.

## Declaration of competing interest

The authors declare that they have no known competing financial interests or personal relationships that could have appeared to influence the work reported in this paper.
